# HIV prevalence and continuum of care among incarcerated people in Iran from 2010 to 2017

**DOI:** 10.1186/s12954-022-00675-9

**Published:** 2022-08-20

**Authors:** Armita Shahesmaeili, Mohammad Karamouzian, Fatemeh Tavakoli, Mostafa Shokoohi, Ali Mirzazadeh, Samira Hosseini-Hooshyar, Saber Amirzadeh Googhari, Nima Ghalekhani, Razieh Khajehkazemi, Zahra Abdolahinia, Noushin Fahimfar, AliAkbar Haghdoost, Hamid Sharifi

**Affiliations:** 1 HIV/STI Surveillance Research Center, and WHO Collaborating Center for HIV Surveillance, Institute for Futures Studies in Health, Kerman University of Medical Sciences, Haft Bagh Highway, Kerman, 7616913555 Iran; 2 Centre On Drug Policy Evaluation, St. Michael’s Hospital, Toronto, ON M5B 1W8 Canada; 3 Dalla Lana School of Public Health, University of Toronto, Toronto, Canada; 4 Department of Epidemiology and Biostatistics, University of California San Francisco, San Francisco, CA USA; 5 The Kirby Institute, UNSW Sydney, Sydney, NSW Australia; 6 Social Determinants of Health Research Center, Institute for Futures Studies in Health, Kerman University of Medical Sciences, P.O. Box 7616913555, Kerman, Iran; 7 Osteoporosis Research Center, Endocrinology and Metabolism Clinical Sciences Institute, Tehran University of Medical Sciences, Tehran, Iran

**Keywords:** HIV, Cascade, Prisoner, Prevalence, Iran

## Abstract

**Background:**

Incarcerated people are at an increased risk of contracting HIV and transmitting it to the community post-release. In Iran, HIV epidemics inside prisons were first detected in the early 1990s. We assessed the HIV prevalence and its correlates, as well as the continuum of care among incarcerated people in Iran from 2010 to 2017.

**Methods:**

We used data collected in three national bio-behavioral surveillance surveys among incarcerated individuals in 2010 (*n* = 4,536), 2013 (*n* = 5,490), and 2017 (*n* = 5,785) through a multistage cluster sampling approach. HIV was tested by the ELISA method in 2010 and 2013 surveys and rapid tests in 2017. Data on demographic characteristics, risky behaviors, HIV testing, and treatment were collected via face-to-face interviews. HIV prevalence estimates along with 95% confidence intervals (CI) were reported. Using data from the 2017 round, multivariable logistic regression models were built to assess the correlates of HIV sero-positivity and conduct HIV cascade of care analysis.

**Results:**

The HIV prevalence was 2.1% (95% CI: 1.2%, 3.6%) in 2010, 1.7% (95% CI: 1.3%, 2.1%) in 2013, and 0.8% (95% CI: 0.6%, 1.1%) in 2017 (trend *P* value < 0.001). Among people with a history of injection drug use, HIV prevalence was 8.1% (95% CI: 4.6%, 13.8%) in 2010, 6.3% (95% CI: 4.8%, 8.3%) in 2013, and 3.9% (95% CI: 2.7%, 5.7%) in 2017. In 2017, 64% (32 out of 50) of incarcerated people living with HIV were aware of their HIV status, of whom 45% (9 out of 20) were on antiretroviral therapy, and of whom 44% (4 out of 9) were virally suppressed (< 1000 copies/ml).

**Conclusions:**

While HIV prevalence has decreased among incarcerated people in Iran, their engagement in the HIV continuum of care is suboptimal. Further investments in programs to link incarcerated people to HIV care and retain them in treatment are warranted.

## Introduction

Incarcerated populations are at a higher risk of contracting human immunodeficiency virus (HIV) [1]; however, HIV sero-positivity estimates among incarcerated people differ significantly across the globe. HIV prevalence among incarcerated populations ranges from < 1 to 1.7% in Asia and Pacific, 0.6%-3.2% in Latin America and the Caribbean, 0.4%-7.5% in Western and Central Europe and North America to 2.1%-24.4% in the Middle East and North Africa, and 2.3%-34.9% in Sub-Saharan Africa [2]. The higher prevalence of HIV among incarcerated populations is mainly attributed to the criminalization of drug use and the detention of people who inject drugs (PWID) [3]. Having unprotected sex, multiple partners, homosexual sex, and alcohol or drug use before or during sex are individual-level factors that put incarcerated individuals at a higher risk of contracting and transmitting STIs, including HIV [1, 2]. Furthermore, overcrowding, poor living conditions, poor access to healthcare services, and delay or lack of diagnosis and treatment contribute to HIV epidemics inside prison settings [3].

In Iran, HIV outbreaks were first noticed among incarcerated people in the mid-1990s [4, 5]. As a response, Iran’s Judiciary system initiated the harm reduction program inside prisons in collaboration with the Ministry of Health in the early 2000s [6]. These harm reduction programs were implemented in the so-called ‘triangular clinics’ and primarily focused on HIV educational interventions, needle and syringe exchange programs (NSPs) for PWID, provision of opioid agonist therapy (OAT) for people with opioid use disorders, and voluntary counseling and HIV testing [4, 7]. HIV testing was initially available only through dried blood testing; however, it is now provided via rapid HIV tests followed by confirmatory laboratory-based analyses. Although the NSPs was associated with reductions in shared needle and syringe practices among incarcerated PWID, it was later discontinued due to prison staff’s workplace health and safety concerns.

Harm reduction services were rapidly scaled up across primary prison settings in all provinces [8], and the number of incarcerated people receiving OAT increased from 100 in 2002 to 25,000 in 2008 and 62,000 in 2019 [4, 9]. However, the quality of harm reduction provision inside prisons remains unclear, and data are limited on the actual coverage of services. For example, around 50% of prisons have an OAT waitlist, and most OAT-related services face space, specialized staff, and budget restrictions; issues that highlight the limited capacity of prisons for timely treatment initiation for people with opioid use disorder [8]. Moreover, care provision in triangular clinics is based on a passive case-finding approach, leading to unmet needs among incarcerated people who engage in HIV-related high-risk practices. Nonetheless, the provision of OAT has been associated with reducing HIV incidence among incarcerated people in a mathematical model [10]; however, causal analyses quantifying the impact of harm reduction interventions on controlling HIV epidemics inside Iranian prisons are lacking. Empirical analyses based on bio-behavioral surveillance surveys have shown that HIV incidence among incarcerated populations has decreased from 1.34 in 2009 to 0.49 per 1,000 in 2013 [11].

The HIV prevention and control programs in Iran aim to meet the 90–90–90 UNAIDS targets and have 90% of all people living with HIV (PLHIV) know their HIV status, 90% of all people with diagnosed HIV infection receive sustained antiretroviral therapy (ART), and 90% of all people receiving ART achieve viral suppression. To reach these ambitious targets and find the potential gaps, regular monitoring of HIV prevalence, especially among key populations, is vital for policymakers. Therefore, in the present study, we assessed the trend and correlates of HIV prevalence and continuum of care among incarcerated people in Iran using the data of three consecutive national bio-behavioral surveillance surveys (BBSS) collected in 2010, 2013 and 2017.

## Methods

### Study design and participants

**Fig. 1:**
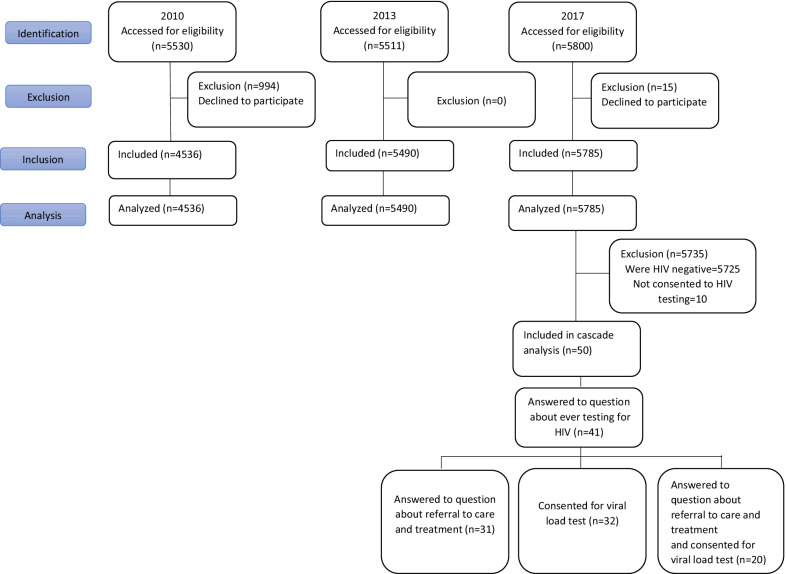
Flow diagram of incarcerated people in Iran recruited in three consecutive bio-behavioral surveillance surveys in 2010, 2013 and 2017

Figure 1 depicts the flow diagram of the study participants and analytical samples in each study round. Out of the 5530, 5511, and 5800 prisoners selected, 4536 (82.0%), 5490 (99.6%), and 5785 (99.7%) incarcerated people consented to HIV testing and participated in the national BBSS in 2010, 2013, and 2017, respectively. These nationwide surveys are conducted every few years to help monitor the trend of HIV and high-risk behaviors among key populations and inform the national HIV response and relevant interventions.

In the first two rounds (2010 and 2013), the study sample was recruited in 27 prisons across 16 provinces, using multistage cluster sampling. We randomly selected 16 provinces and classified the prisons into large (i.e., ≥ 500 incarcerated people) and small prisons (i.e., < 500 incarcerated people). We then selected 14 large and 13 small prisons using a stratified random sampling approach. In the 2017 round, the sampling logic was similar to the sampling of previous rounds, but more prisons (33 prisons) were included in the survey [12]. Those aged 18 years or more who had been incarcerated for at least one week, had not participated in similar studies within the last two months, were Iranian, and provided verbal informed consent for participation in the study were eligible. Both male and female participants were recruited proportional to the size of the population inside each prison.

### Data collection

Data were collected through face-to-face interviews using a structured risk assessment questionnaire. Data were obtained on participants’ sociodemographic characteristics, substance use practices, sexual behaviors, HIV-related knowledge, previous incarceration history, HIV testing, tattooing, substance use treatment, and care-seeking practices inside prisons.

### Dependent variable: HIV sero-status

The outcome variable was HIV sero-status at the time of the study. Sero-positive results were coded as 1 and sero-negative results as 0. In the first two survey rounds, HIV sero-status was determined by taking dried blood spots and testing for anti-HIV-1–2 antibody/antigen via the ELISA method (Vironostika HIV Uni-Form II Ag/Ab, bioMérieux, France). Positive tests of the first ELISA were examined by a confirmatory ELISA developed by a different manufacturer. If the second test was negative, this participant was considered sero-negative. All tests were carried out by a central reference laboratory in Iran’s Ministry of Health. In the third survey round, two rapid HIV tests were used. Participants with a negative result for the first rapid test (SD Bioline HIV-1/2, 3.0) were considered sero-negative. However, individuals with a positive result in the first test underwent a second rapid test (Uni-Gold™ Recombigen® HIV-1/2). If the second test was positive, they were considered HIV sero-positive and referred to a triangular clinic for further evaluation and linkage to treatment. The test results were returned to the participants anonymously using a unique code in all rounds. Pre- and post-test counseling was available and provided in the prison clinic. Interviews and blood sampling were completed in a private room inside the prison.

### Covariates

Covariates of interest were: age at the time of interview (≤ 25 or > 25 years), sex (male or female), marital status (single/never married, currently married, or previously married), education levels (≤ high school or > high school), history of incarceration (yes or no), history of non-injection drugs (yes or no), history of injection drug use (yes or no), age at first drug use (≤ 18 or > 18), age at first injection (≤ 18 or > 18), history of tattooing (yes or no), condom use at last sex (yes or no), history of same-sex practices (yes or no), and condom use at the last homosexual sex (yes or no).

### HIV cascade of care and treatment

Based on the 90–90–90 indicators, the HIV care cascades were examined for incarcerated PLHIV in three steps: 1st 90: Proportion of PLHIV aware of their status, 2nd 90: Proportion of PLHIV aware of their status and on ART, and 3rd 90: Proportion of PLHIV aware of their status, on ART, and virologically suppressed. The nominator and denominator for these indicators are presented in Table 1. The viral suppression was defined as < 1000 copies per milliliter of blood. All tests were completed in a central reference laboratory in Tehran.

**Table 1:** Calculation of HIV treatment cascade indicators among incarcerated people living with HIV in Iran 2017

Cascade indicator	Numerator	Denominator
1st 90: Proportion of HIV-positive participants aware of their status	Total number of participants who reported knowing they were HIV positive	Total number of participants who had a positive HIV test result
2nd 90: Proportion of HIV-positive participants aware of their status and on treatment	Total number of participants who reported being on ART	Total number of participants who had a positive HIV test result and were aware of their status
3rd 90: Proportion of HIV-positive participants aware of their status, on treatment, and virologically suppressed	Total number of participants with virologic suppression (viral load < 1000 copies /ml)	Total number of participants who had a positive HIV test result, were aware of their HIV-positive status, and reported being on ART

### Quality control

To reduce the potential variability in HIV testing procedures across different prisons, we employed experienced staff to conduct interviews and HIV counseling and testing procedures. All staff completed a coordinated hands-on training workshop prior to the study and were provided with a detailed written guideline on data collection procedures. Two weeks after study initiation, the study team visited all the study locations to resolve any potential logistical issues and ensure the data collection guidelines were closely followed.

### Statistical analysis

We reported the prevalence of HIV and its 95% confidence interval (CI) among incarcerated people in the three rounds and compared them using the chi-square test for trend. HIV prevalence was also reported by subgroups of the covariates. Bivariable and multivariable logistic regression models were constructed only for the third round (2017) to assess the correlates of HIV sero-positivity among the participants. Variables with a *P* value < 0.2 from the bivariable models were entered into the multivariable model. The final model was built using the backward elimination approach. As participants were recruited from different prison settings, each setting was considered as a cluster, and their clustering effects were adjusted using Stata’s survey package. The survey weights were calculated by dividing the total population by the sample size of each prison. Crude and adjusted odds ratios (AORs) along with 95% confidence intervals (CI) were reported. Stata version 14.1 (College Station, Texas) was used throughout the data analysis. *P* values < 0.05 were considered statistically significant.

## Results

### Participants’ characteristics

In all surveys, most participants were male (95.6% in 2010, 98.1% in 2013 and 94.0% in 2017), had an education level less than high school (96.2% in 2010, 95.0% in 2013 and 92.9% in 2017), were > 25 years (75.5% in 2010, 87.8% in 2013 and 88.1% in 2017), and were married (53.6% in 2010, 54.5% in 2013 and 50.8% in 2017).

Overall, the prevalence of HIV showed a significant decreasing trend from 2.1% (95% CI: 1.2%, 3.6%) in 2010, to 1.7% (95% CI: 1.3%, 2.1%) in 2013, and 0.8% (95% CI: 0.6, 1.1) in 2017 (*P* value of trend test < 0.001). Among prisoners who reported a history of injection drug use, the HIV prevalence decreased from 8.1% (95% CI: 4.6%, 13.8%) in 2010 to 6.3% (95% CI: 4.8%, 8.3%) in 2013 to 3.9% (95% CI: 2.7, 5.7) in 2017. Furthermore, in all studied subgroups, HIV prevalence decreased. However, it was more prominent among prisoners who were male (2.1% in 2010, 1.7% in 2013 and 0.8% in 2017; *P* value < 0.001), aged > 25 years (2.2% in 2010, 1.8% in 2013 and 0.8% in 2017; *P* value < 0.001), were educated up to high school (2.0% in 2010, 1.8% in 2013 and 0.9% in 2017; *P* value < 0.001), were never married (2.6% in 2010, 2.7% in 2013 and 0.9% in 2017; *P* value = 0.002) or were previously married (4.2% in 2010, 3.1% in 2013 and 0.7% in 2017; *P* value < 0.001), had ever used non-injecting drugs (2.5% in 2010, 2.0% in 2013 and 0.9% in 2017; *P* value < 0.001), had never injected drugs (0.9% in 2010, 0.7% in 2013 and 0.3% in 2017; *P* value < 0.001), and had a history of tattooing (3.3% in 2010, 2.6% in 2013 and 1.2% in 2017; *P* value < 0.001) (Table 2).

**Table 2:** HIV prevalence among incarcerated people in Iran in three consecutive bio-behavioral surveillance surveys in 2010, 2013, and 2017

Variables	2010			2013			2017			Trend
	*N* (%)	HIV Prevalence (95% CI)	*P* value	*N* (%)	HIV Prevalence (95% CI)	*P* value	*N* (%)	HIV Prevalence (95% CI)	*P* value	*P* value
Overall prevalence	4536	2.1 (1.2, 3.6)	–	5490	1.7 (1.3, 2.1)	–	5785	0.8 (0.6,1.1)	–	< 0.001
*Age*										
≤ 25	1111 (24.5)	1.3 (0.7, 2.1)	0.111	670 (12.2)	0.9 (0.4, 2.4)	0.181	689 (11.9)	0.4 (0.1, 1.5)	0.359	0.061
> 25	3421 (75.5)	2.2 (1.7, 2.7)		4820 (87.8)	1.8 (1.4, 2.3)		5091 (88.1)	0.8 (0.6, 1.1)		< 0.001
*Sex*										
Male	4337 (95.6)	2.1 (1.9, 3.6)	0.910	5387 (98.1)	1.7 (1.3, 2.1)	0.401	5435 (94.0)	0.8 (0.6, 1.0)	0.916	< 0.001
Female	199 (4.4)	1.9 (0.6, 5.9)		103 (1.9)	0.0 (0.0, 3.5)		350 (6.0)	0.9 (0.2, 5. 9)		0.062
*Level of education*										
High school or less	4360 (96.2)	2.0 (1.7, 2.5)	0.187	5204 (95.0)	1.8 (1.4, 2.2)	0.231	5361 (92.9)	0. 9 (0.7, 1.2)	0.011	< 0.001
More than high school	172 (3.8)	0.6 (0.09, 4.4)		277 (5.0)	0.8 (0.2, 3.01)		411 (7.1)	0.01 (0.001, 0.7)		0.430
*Marital status*										
Never married	1663 (36.9)	2.6 (1.9, 3.5)	< 0.001	1825 (33.2)	2.7 (1.99, 3.7)	< 0.001	1915 (33.1)	0.9 (0.6, 1.5)	0.548	0.002
Currently married	2415 (53.6)	1.08 (0.7, 1.6)		2998 (54.5)	0.7 (0.5, 1.1)		2939 (50.8)	0. 7 (0.4, 1.1)		0.091
Previously married	428 (9.5)	4.2 (2.7, 6.6)		661 (12.3)	3.1 (1.8, 5.3)		928 (16.1)	0.7 (0.3, 1.6)		< 0.001
*Incarceration history*										
Yes	1872 (46.4)	2.9 (2.2, 3.7)	< 0.001	3260 (59.4)	2.4 (1.9, 3.1)	< 0.001	3419 (59.1)	0.9 (0.7, 1.4)	0.080	< 0.001
No	2166 (53.6)	1.25 (0.86,1.81)		2228 (40.6)	0.58 (0.32, 1.07)		2362 (40.9)	0.48 (0.23, 1.01)		0.001
*Ever had non-injection drug use*										
Yes	3330 (73.5)	2.5 (2.0, 3.1)	< 0.001	4367 (79.6)	2.0 (1.6, 2.6)	0.002	4424 (76.5)	0.9 (0.7, 1.3)	0.094	< 0.001
No	1200 (26.5)	0.4 (0.2, 1.0)		1119 (20.4)	0.4 (0.1, 1.2)		1360 (23.5)	0.2 (0.0, 1.3)		0.173
*Ever had drug injection*										
Yes	726 (16.0)	8.1 (4.6, 13.8)	< 0.001	991 (22.7)	6.3 (4.8, 8.3)	< 0.001	705 (12.2)	3.9 (2.7, 5.7)	< 0.001	0.081
No	3803 (84.0)	0.9 (0.5, 1.6)		3368 (77.3)	0.7 (0.4, 1.0)		5065 (87.8)	0.3 (0.2, 0.5)		< 0.001
*Age at first drug use*										
≤ 18	1399 (42.6)	3.5 (2.6, 4.6)	0.001	1945 (44.5)	2.9 (2.2, 3.9)	< 0.001	2059 (46.9)	1.1 (0.7, 1.7)	0.253	< 0.001
> 18	1884 (57.4)	1.7 (1.2, 2.4)		2422 (55.5)	1.2 (0.8, 1.9)		2329 (53.1)	0.8 (0.5, 1.3)		0.004
*Age at first injection*										
≤ 18	113 (15.9)	12.4 (7.4, 19. 9)	0.036	127 (12.8)	11.3 (6.3, 19.2)	0.031	99 (14.3)	5.3 (2.2, 12.3)	0.487	0.182
> 18	598 (84.1)	6.7 (4.9, 9.0)		864 (87.2)	5.57 (4.06, 7.61)		594 (85.7)	3.8 (2.5, 5.7)		0.301
*History of tattooing*										
Yes	2041	3.3 (2.1, 5.4)	< 0.001	2672 (48.7)	2.6 (1.9, 3.4)	< 0.001	2733 (47.3)	1.2 (0.8, 1.7)	0.004	< 0.001
No	2492	1.0 (0.5, 2.2)		2815 (51.3)	0.8 (0.5, 1.3)		3051 (52.7)	0.4 (0.2, 0.8)		0.082
*Condom use at last sex*										
Yes	–	–		1144 (24.5)	2.0 (1.2, 3.2)	0.307	1267 (25.4)	0. 9 (0.5, 1.6)	0.614	0.214
No	–	–		3532 (75.5)	1.5 (0.9, 2.0)		3725 (74.6)	0.7 (0.5, 1.1)		0.060
*History of same-sex practices*										
Yes	–	–		562 (12.1)	2.9 (1.8, 4.9)	0.012	714 (14.7)	1.1 (0.6, 2.2)	0.244	0.104
No	–	–		4085 (87.9)	1.4 (1.0, 1.9)		4143 (85.3)	0.7 (0.5, 1.0)		0.141
*Condom use at last same-sex practice*										
Yes	–	–		33 (6.0)	7.3 (1.8, 24.9)	0.174	36 (5.1)	0	0.515	0.128
No	–	–		521 (94.0)	2.7 (1.6, 4.7)		670 (94.9)	1.2 (0.6, 2.4)		0.210

### Correlates of HIV sero-positivity

In the multivariable model based on the 2017 data, the only variable that was significantly associated with the increased odds of HIV sero-positivity was ever injecting drugs (AOR = 13.79; 95% CI: 6.85, 27.75, *P* value < 0.001) (Table 3).

**Table 3:** Correlates of HIV sero-positivity among incarcerated people in Iran in 2017, using multivariable logistic regression analysis

Variable	Crude OR (95% CI)	*P* value	Adjusted OR (95% CI)	*P* value
*Level of education*				
High school or less	1	0.034	–	–
More than high school	0.12 (0.02, 0.85)		–	
*Incarceration history*				
No	1	0.086	–	–
Yes	2.04 (0.90, 4.62)		–	
*Ever had non-injection drug use*				
No	1	0.121	–	–
Yes	3.98 (0.70, 22.76)		–	
*Ever drug injection*				
No	1	< 0.001	1	< 0.001
Yes	13.79 (6.85, 27.75)		13.79 (6.85, 27.75)	
*History of tattooing*				
No	1	0.006	–	–
Yes	2.73 (1.33, 5.61)		–	

### HIV cascade of care and treatment

**Fig. 2:**
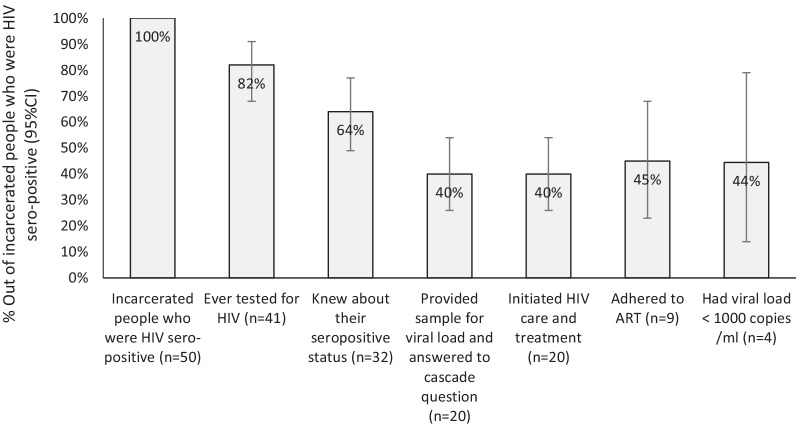
HIV sero-status awareness and treatment cascade among incarcerated people living with HIV in Iran (2017)

1st 90: Proportion of PLHIV aware of their status: Of 50 incarcerated PLHIV (using data from the 2017 survey), only 41 individuals answered the question about ever testing for HIV. Of them, 32 participants (64% [95% CI: 49, 77]) knew that they were HIV sero-positive. 2nd 90: Proportion of PLHIV aware of their status and on ART treatment: Of the whole PLHIV (*n* = 50), only 20 participants answered the questions about their referral to HIV care and treatment. All of them were aware of their HIV sero-positivity. Of them, 45% (95% CI: 23, 68) (*n* = 9) were currently on ART. 3rd 90: Proportion of PLHIV aware of their status, on ART, and virologically suppressed: 4 out of 9 participants who were currently on ART (44% [95% CI: 14, 79]) reached viral suppression (Fig. 2).

## Discussion

Our findings suggested a decrease in HIV prevalence from 2.1% in 2009 to 0.8% in 2017 among incarcerated people in Iran. Although the HIV prevalence among incarcerated PWID decreased from 8.1% to 3.9%, it was up to 13 times higher than those without a history of injection drug use. One in five incarcerated PLHIV were unaware of their HIV status, and more than half were not on ART. Moreover, less than half of incarcerated PLHIV on ART were virally suppressed. Given the absence of precise and reliable data, comparing the obtained HIV prevalence estimates among incarcerated people in our study with those in international settings is challenging. However, our estimates among incarcerated people in Iran suggest a lower HIV prevalence when compared to pooled regional estimates among incarcerated people as reported in a meta-analysis combining data from 1980 to 2017 (e.g., 3% in Asia, 4% in North America, 5% in Europe, and 6% in Africa) [13].

Our findings suggest a significant decrease in HIV prevalence among incarcerated people since the 1990s’ HIV outbreak inside several prison settings [14]. This decrease could be partly explained by the decline in HIV incidence among incarcerated people, as outlined in our previous study in Iran suggesting a decrease in HIV incidence among incarcerated people from 1.34 (2009) to 0.49 (2013) per 1000 person-year [11]. One of the potential explanations for this decline might be the expansion of harm reduction programs (e.g., scaling up of OAT, NSPs, voluntary HIV testing, and ART) inside prisons and in the broader community [15]. Indeed, the provision of OAT has been associated with reducing HIV incidence among incarcerated people in a mathematical model [16], and NSPs operating in a few prison settings have led to significant reductions in the average number of a shared needle per week from 3.7 to zero [13]. However, causal analyses quantifying the impact of harm reduction interventions on controlling HIV epidemics inside Iranian prisons are lacking and warranted.

Our findings that HIV prevalence was highest among incarcerated people with a history of injection drug use are concerning but expected given the established HIV epidemics among PWID in Iran. While some PWID reduce or cease their injection drug use practices during incarceration, international evidence suggests that a large proportion of PWID continue to inject while incarcerated and post-release, and a smaller group even initiate injection drug use inside prisons [17, 18]. This is particularly important in the context of Iran, where despite the decreasing prevalence of HIV (from 15.1% in 2010 to 3.5% in 2020), self-reported unsafe injections practices have increased, particularly among PWID who were living with HIV (53.1% vs. 33.9% among HIV sero-negative PWID) [19] and most incarcerated people reported a lifetime history of substance use [20]. Moreover, about one-eighth of the study population in 2017 had a history of injection drug use. These collective observations call for revisiting existing harm reduction services for PWID and reintroducing the previously discontinued NSPs inside prisons, reforms that could follow the international experiences across different low- and high-income settings that have successfully implemented various forms of NSPs operation models inside prisons (e.g., direct distribution by health staff, peer-led distribution, and automated dispensing machines) [18, 21]. Additionally, as a considerable proportion of incarcerated people in Iran—ranging from 45% to 98.5% across different studies—are incarcerated due to drug-related charges [22–25], there is a need to reduce the rate of prison entry due to non-violent drug-related offences [26].

Although most incarcerated PLHIV were aware of their status, more than half of PLHIV (55%) who knew about their status were not on ART. These estimates are higher than the 2014 national HIV cascade of care estimates among all PLHIV in Iran—where 30% of PLHIV were diagnosed, and 8% had received ART [27]—primarily due to the provision of HIV testing and treatment services inside prisons [28]. Conversely, less than half (44%) of incarcerated PLHIV who were on ART had reached viral suppression, which is lower than the national estimates (i.e., 85%) [29]. This is concerning given that viral load suppression is a proxy for ART adherence and helps avoid an array of adverse health outcomes, such as the development of ART resistance, progression to AIDS, and death [30]. These findings align with the pooled ART adherence estimates among incarcerated people in a global systematic review of the evidence reported from 1998 to 2013 (i.e., 54.6%; 95% CI: 48.1–60.9) [31]. While we did not explore the underlying reasons for low ART adherence among incarcerated people in Iran, previous evidence from Iran has associated insufficient ART adherence among incarcerated people with several factors, such as psychological disorders, poor physical condition, unpleasant prison setting, HIV-related stigma, lack of family support, substance use disorders, and poor adherence to OAT [32].

Overall, the derived HIV cascade of care estimates highlight a significant gap in reaching the UNAIDS 90–90–90 targets and call for Iran’s further investments in active routine HIV counseling and testing, and confidential HIV care and treatment provision inside prison settings, as well as peer-led education and linkage to care post-release [33, 34]. While post-release services, such as linkages to community-based services to ensure OAT and ART continuity, are available in Iran, their effectiveness is understudied and quite low based on the limited information available (e.g., only one-third of PLHIV who are newly released from prison are appropriately linked to HIV care and treatment post-release) [23].

## Limitations

We acknowledge the limitations of our study. First, our findings are prone to reporting and social desirability biases. Indeed, a considerable body of evidence suggests that PLHIV face multi-layered HIV-related stigma across various social and healthcare settings that may lead to reluctance in disclosing their HIV status or seeking HIV-related care and treatment [35–38]. Experiences of HIV-related stigma are often more pronounced in the closed settings of prisons, where PLHIV face negative attitudes and discrimination from their fellow incarcerated people and prison staff [39, 40]. Although we tried to conduct the interviews in a private room inside prisons and employed experienced local staff to collect data, such biases cannot be ruled out. Second, the study’s design was cross-sectional and prone to temporality bias. Third, our assessment of the first 90 of the UNAIDS 90–90–90 targets (i.e., awareness of HIV-positive status) via self-report is subject to underreporting. Moreover, some participants did not consent to the viral suppression test or answer questions related to the HIV cascade of care and ART adherence, which could have affected the validity and precision of our estimates. Lastly, the method of HIV screening across the three study rounds was not similar, which might have impacted the comparability of findings over time. However, the utilized HIV screening approaches were reliable and approved by a central reference laboratory.

## Conclusions

The decreasing trend of HIV prevalence among incarcerated people in Iran is promising and might be partly explained by the scale-up of harm reduction services across prison settings and in the broader community. However, despite the relatively high rate of HIV awareness among Iranian prisoners, engagement in treatment and virological suppression remains suboptimal and significant gaps exist in accessing and adhering to ART among incarcerated people in Iran. Further investments in how HIV care and treatment are provided to incarcerated people during incarceration and post-release are warranted.

## Data Availability

The datasets used and/or analyzed during the current study are available from the corresponding author upon reasonable request.
